# Modeling ventilation heterogeneity in lung fibrosis

**DOI:** 10.1007/s11517-026-03536-w

**Published:** 2026-04-01

**Authors:** Irina Tirsina, Raffaella Rizzoni, Roberto Tonelli, Alessandro Marchioni, Maria Letizia Raffa

**Affiliations:** 1 Laboratoire EULER, ISAE-Supméca, 3 Rue Fernand Hainaut, Saint-Ouen-Sur-Seine, 3400 France; 2 Department of Engineering, University of Ferrara, Via Saragat 1, Ferrara, 44122 Italy; 3 Respiratory Diseases Unit, Department of Medical and Surgical Sciences, University Hospital of Modena, University of Modena Reggio Emilia, Via del Pozzo 71, Modena, 41124 Italy

**Keywords:** Idiopathic pulmonary fibrosis, Computational model, Alveolar mechanics, Pulmonary ventilation

## Abstract

Mechanical forces arising from tissue heterogeneity are thought to influence the progression of idiopathic pulmonary fibrosis (IPF), but their impact under physiological variability remains unclear. We developed a multi-scale computational model of the human lung by embedding a micromechanical alveolar model within a gravitationally stratified lung model. Physiological variability was incorporated through a Monte Carlo uncertainty quantification of material and geometric parameters derived from clinical and literature data. Simulations were performed for three scenarios: healthy lung (H), uniformly fibrotic lung (UIPF), and heterogeneously fibrotic lung with a stiff base and compliant apex (SWIPF). The model’s predictions of macroscopic lung volumes showed strong quantitative agreement with clinical data. Notably, the predicted mean total lung capacity (TLC) for the SWIPF model (3.89 L) differed from the clinical mean for IPF patients (3.95 L) by only 1.5%. Importantly, only the heterogeneous SWIPF model reproduced the characteristic non-uniform ventilation pattern observed in CT scans of IPF patients, achieving a Normalized Root Mean Square Error (NRMSE) of 19.75% relative to the clinical data. This represented a significant improvement over the UIPF model, which had an NRMSE of 30.25%. These results demonstrate that structural heterogeneity is the key determinant of ventilation patterns in IPF and highlight mechanically vulnerable regions that could contribute to disease progression.

- A multi-scale model of IPF lung mechanics is developed and validated.
- Model predictions of lung volumes match clinical population statistics.
- Heterogeneous collagen distribution reproduces patient ventilation patterns.
- Hyperinflation is predicted in preserved tissue near fibrotic regions.

**Graphical Abstract:**

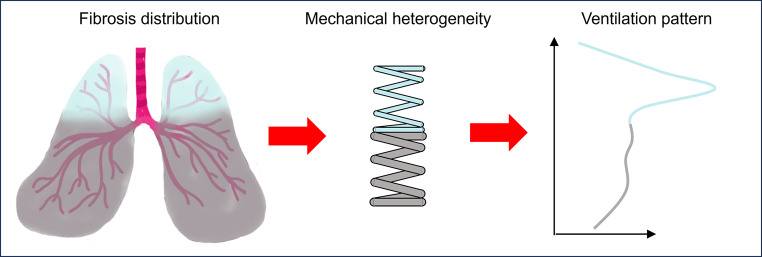

## Introduction

Idiopathic pulmonary fibrosis (IPF) is a progressive lung disease that leads to scarring lung tissue and a severe reduction in lung function. Patients typically survive only three to five years after diagnosis, and current treatments offer limited benefit [1, 2]. A hallmark of IPF is the excessive deposition of extracellular matrix (ECM), which stiffens the lung and impairs gas exchange [1]. While several risk factors, such as smoking, environmental exposures, and genetic variants, have been identified, the exact mechanisms that drive progressive lung damage remain unclear [2].

Traditionally, research on IPF has focused on biochemical pathways. However, there is increasing evidence that mechanical factors are crucial in disease progression [3–6]. The “mechanical vicious cycle” hypothesis suggests that fibrotic regions create local stiffness heterogeneity. During breathing, this causes excessive deformation at the interfaces between stiff and compliant tissue, activating pro-fibrotic cellular pathways such as TGF-$\beta$ 1 signaling and HOXB7 expression [7–10]. This feedback loop further promotes ECM deposition and tissue stiffening.

Computational modeling has been widely applied to explore mechanobiological processes in the lung. Recent review articles discuss different approaches and their potential to capture interactions between tissue mechanics, cellular behavior, and fibrosis progression [5, 11–15]. At the alveolar scale, several computational models, including agent-based models (ABMs), network, and hybrid physics/ABM approaches, explore how fibroblasts respond to changes in ECM stiffness and strain, and how these responses may drive local fibrosis. For example, Hall et al. used an agent-based spring network model to demonstrate the roles of both stiffness and stretch in fibrotic pattern development [16]. Earlier work by Oliveira, Bates, and Suki explored spatially correlated collagen deposition in bidimensional networks and its impact on bulk tissue rigidity [17]. Wellman et al. developed a hybrid physics/agent-based model to explain the formation of honeycomb patterns in IPF via mechanically driven fibroblast activation [18]. These models collectively highlight mechanobiological feedback as a key factor in fibrosis evolution. At the organ scale, continuum models have been used to describe the elastic response of lung tissue, capturing non-linear and anisotropic behaviors predicted by micromechanical simulations, cf. [5, 11, 19, 20]. These models provide a framework to represent the lung as a deformable solid and to study tissue mechanics under various loading conditions. Poromechanical models extend this approach by coupling the tissue matrix with the airways, allowing simulation of regional ventilation patterns, alveolar pressure distribution, and tissue stress [21–24]. Some poromechanical models have been personalized using patient imaging data to estimate regional lung compliances and assess the impact of fibrosis on tissue stiffening [ 25, 26]. Other studies have incorporated gravity to explore its effects on local tissue dimensions, pressure–volume response, and stress distribution [27]. Additional work has included dynamic surface tension and collagen viscoelasticity to investigate their impact on the hysteresis loop of the lung P–V curve and regional ventilation [28].

Although progress has been made at both micro- and organ-level scales, establishing quantitative links between micromechanical behavior and patient-level outcomes under physiological variability remains an area of active research. To advance this direction, we propose a multi-scale computational model of the human lung that combines a validated micromechanical alveolar model [29] with a multi-layer representation of lung tissue to account for vertical gradients in pleural pressure and mechanical stress proposed by Shi et al. [28]. We use this model to predict macroscopic lung volumes and regional ventilation in healthy and fibrotic lungs. By incorporating an inhomogeneous distribution of material properties along the caudal-cranial axis, our approach allows exploration of how micro-scale differences may influence whole-lung mechanics. Validation against clinical population data and patient-specific CT measurements demonstrates that this modeling approach provides a novel quantitative framework for linking micro-scale mechanical variations to lung function in IPF, which may guide future multi-scale studies of lung mechanics and fibrosis progression.

## Methods

### Theoretical model

Here, we summarize the alveolar model proposed in [29]. We then combine this model with the approach proposed in [28] to investigate the effect of gravity on lung deformation.

#### Alveolar model

In [29], the alveolus is modeled as a spherical shell composed of alternating layers of elastin and collagen. The shell is initially stress-free and subjected to a given (transalveolar) pressure $P.$ The thin membrane approximation yields a nonlinear relationship between the inflating pressure and the circumferential stretch, $\lambda ,$ cf. [45, Eq. (15), Add. Inform.1.

1 $$P=\epsilon \sum\nolimits_{j=1}^{2}{\chi }_{j}\frac{{\dot{w}}^{(j)}(\lambda )}{{\lambda }^{2}},$$

where $\epsilon$ is the ratio of total thickness to internal radius of the membrane, ${\chi }_{1}$ and ${\chi }_{2}$ represent the volume fractions of elastin and collagen in alveolar walls, respectively, and ${\dot{w}}^{(j)},j=\mathrm{1,2},$ denote first derivatives of the functions ${w}^{(j)},j=\mathrm{1,2},$ related to the strain energy densities of elastin and collagen. Both elastin and collagen are assumed to be nonlinear hyperelastic, isotropic and incompressible materials, with strain energy densities denoted ${W}^{(1)}$ and ${W}^{(2)},$ respectively. These energy densities are assumed to take the form

2 $${W}^{(1)}({\lambda }_{r},{\lambda }_{\theta },{\lambda }_{\phi })={c}_{1}({\lambda }_{r}^{2}+{\lambda }_{\theta }^{2}+{\lambda }_{\phi }^{2}-3),$$

3 $${W}^{(2)}({\lambda }_{r},{\lambda }_{\theta },{\lambda }_{\phi })={c}_{2}({\lambda }_{r}^{2}+{\lambda }_{\theta }^{2}+{\lambda }_{\phi }^{2}-3{)}^{3},$$

where ${\lambda }_{r},{\lambda }_{\theta }$ and ${\lambda }_{\phi }$ are three principal stretches in the radial, meridian and circumferential directions, respectively, consistently with the assumption of axial symmetry for the spherical membrane, and ${c}_{1},{c}_{2}$ are material parameters governing the stiffness contributions of the elastin and collagen networks, respectively. The functions ${w}^{(j)},j=\mathrm{1,2},$ are defined such that

4 $${w}^{\left(j\right)}\left(\zeta \right)={W}^{\left(j\right)}\left({\zeta }^{-2},\zeta ,\zeta \right), j=1,2.$$

The circumferential stretch, $\lambda ,$ is related to the alveolar volume, $V,$ as follows

5 $$V={V}_{0}{\lambda }^{3},$$

where ${V}_{0}$ denotes the initial alveolar volume, i.e. the alveolar volume at $P=0.$ Given the transveolar pressure $P,$ the circumferential stretch $\lambda$ can be calculated from Eq. (1), and using Eq. (5) the alveolar volume, $V,$ can be computed.

#### Gravitational model

**Fig. 1:**
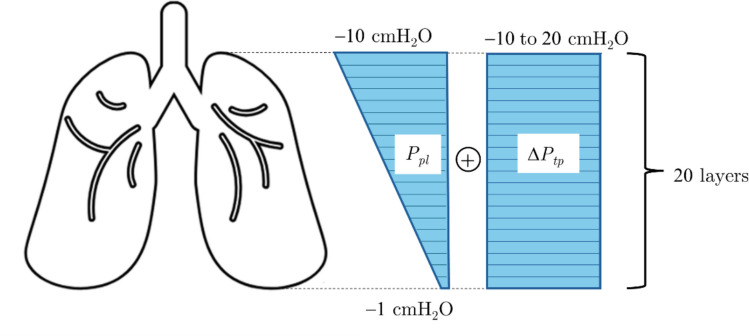
Schematic representation of the upright human lung, illustrating the linear gradient of pleural pressure ${P}_{pl}$ and the applied pressure during breathing, $\Delta {P}_{tp}$ [28]

For our gravitational model, we adopted the 20–layer cranio-caudal discretization of the upright human lung proposed by Shi et al. [28] and schematized in Fig. 1. This subdivision allows to account for the effects of gravity, which influences the distribution of alveolar volumes across the different regions of the lung. At functional residual capacity (FRC), the pleural pressure ${P}_{pl}$ is prescribed with a linear gradient, ranging from $-1$ cmH_2_O at the base to $-10$ cmH_2_O at the apex, reflecting gravitational effects on the pleural pressure. The local transalveolar pressure in each region is defined as

6 $${P}_{ta,i}={P}_{alv,i}-{P}_{pl,i}, i=\mathrm{1,2},\dots 20,$$

where $i=1$ corresponds to the base and $i=20$ to the apex. Under static conditions, the pressure within the alveolar pressure is assumed to be equal to the pressure at the airway entry point (the mouth). Airflow and temporal acceleration of the gas are neglected, implying that ${P}_{alv,i},i=\mathrm{1,2},\dots 20,$ is equal to atmospheric pressure, which is set to a reference of $0$ cmH_2_O. Thus

7 $${P}_{ta,i}=-{P}_{pl,i}, i=\mathrm{1,2},\dots 20.$$

**Fig. 2:**
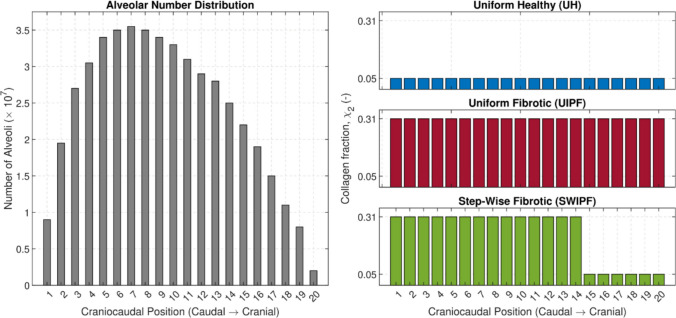
Spatial distributions used as model inputs. The left panel shows the distribution of alveolar numbers along the cranio-caudal axis adapted from [28] using WebPlotDigitizer [30]. The right panel illustrates the three collagen volume fraction distributions investigated: a healthy uniform distribution (UH, ${\chi }_{2}=0.05$), a uniform fibrotic distribution (UIPF, ${\chi }_{2}=0.31$), and a stepwise fibrotic distribution (SWIPF) modeling a sharp gradient between fibrotic basal and preserved apical regions

Using this pressure distribution and the pressure–volume response given by Eq. (1), region-specific alveolar volume changes at FRC, denoted as ${V}_{FRC,i}, i=\mathrm{1,2},\dots 20,$ are determined. The regional volume is then determined by multiplying the alveolar volume for the regional number of alveoli, ${N}_{i}.$ The distribution of alveolar numbers along the cranio-caudal axis is represented in the left panel of Fig. 2.

The total lung volume at FRC, ${V}_{FRC,tot},$ is then computed by summing the volumes from all $i$ cranio-caudal layers. Lung inflation from FRC to total lung capacity (TLC) is simulated by adding an increment in transpulmonary pressure $\Delta {P}_{tp},$ which is positive during inflation and negative during deflation.

**Fig. 3:**
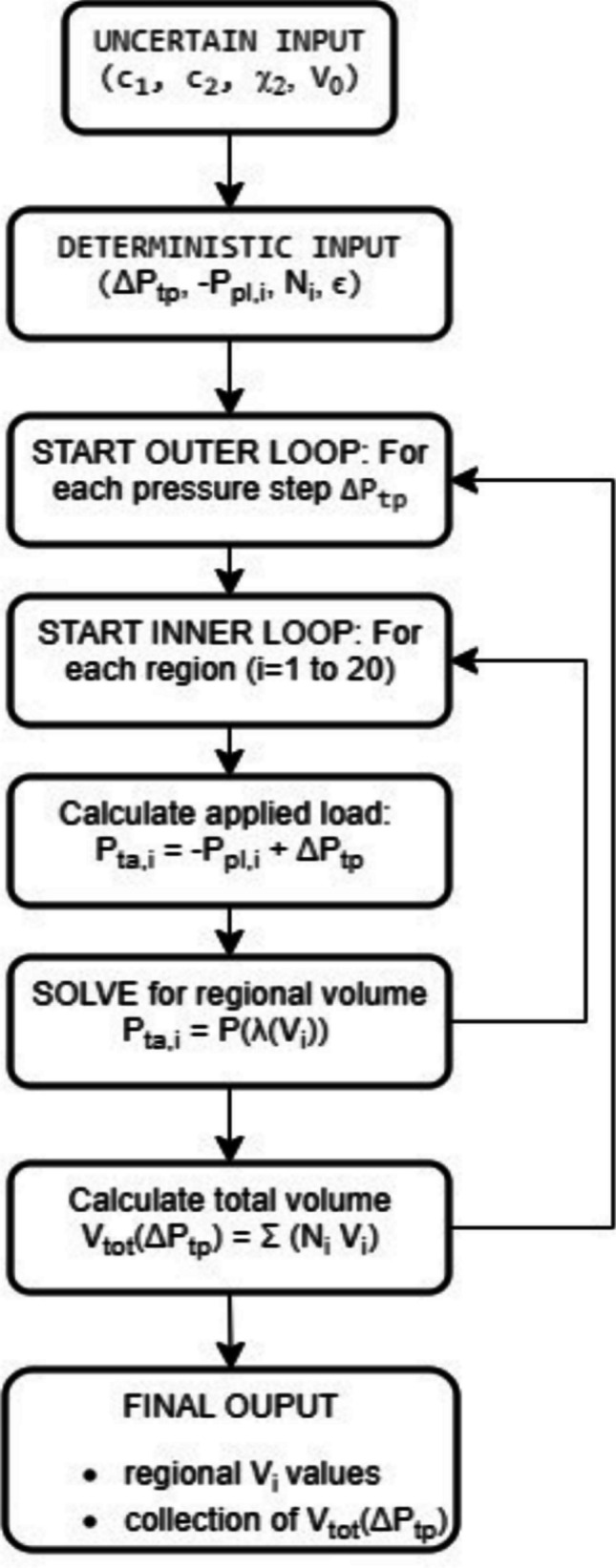
Flowchart of the procedure adopted from [28] to compute the regional volume distribution and the total lung volume at a given level of the applied pressure during breathing, $\Delta {P}_{tp}$

The incremental pressure $\Delta {P}_{tp}$ is assumed to be uniformly distributed from the apex to the base of the lungs. For each prescribed transpulmonary pressure step, $\Delta {P}_{tp},$ a procedure like the one used at FRC is used to calculate the alveolar, regional and global volumes, as schematized in the flowchart shown in Fig. 3.

In [28], an alveolar model different from the one presented in the previous Section is used to compute alveolar inflation volumes across lung layers. Viscoelasticity and surfactant effect were also considered. Here, we neglect viscoelasticity and adopt the alveolar model based on Eq. (1). As will be discussed in the limitations, this model also implicitly includes the effects of surfactant in its material parameters rather than modeling them explicitly. Finally, we assume the alveolar model based on Eq. (1) valid only for positive strain values $\frac{\Delta V}{{V}_{0}}$. Specifically, when combined with the gravitational model, negative pressure values acting on regional alveolar membranes at residual volume (RV) are set to correspond to unitary stretch. This approach aligns with the methodology proposed by Shi et al. in [28] to avoid negative volume changes.

### Clinical data and image analysis

Pulmonary function test (PFT) and CT data were derived from a previously published cohort of 20 adult patients with IPF and 15 healthy controls [31]. Inclusion criteria were age ≥ 18 years and availability of complete lung volume measurements. Demographic characteristics of the cohorts have been previously reported [31]. IPF patients were predominantly older adults (median age 78 years), mainly male and former smokers, while healthy controls were younger (median age 68 years) with normal pulmonary function and no evidence of lung disease. Mean ± SD at TLC was 3.95 ± 0.60 L in IPF patients and 5.88 ± 0.66 L in healthy controls; RV was 1.46 ± 0.38 L and 2.06 ± 0.47 L, respectively. These data were used for global model parameterization and validation.

CT-based analysis was performed on a subset of six IPF patients from the same cohort, selected according to strict image quality criteria. This subset included four males and two females, with a median age of 68 years; all patients were former smokers. Inclusion criteria for CT analysis were the availability of volumetric chest CT acquired at TLC and RV using a standardized protocol, absence of motion artifacts, and suitability for reliable lung segmentation. CT scans were acquired during a single full inspiratory breath-hold at TLC and a forced expiratory breath-hold at RV, with patients in the supine position.

The scan analysis was conducted using two software applications. Initially, the images were imported into Bee DICOM, a specialized platform for managing and processing medical imaging data [32]. Using Bee DICOM, we extracted the files corresponding to lungs at TLC and RV, which served as the basis for subsequent analyses.

These files were then imported into 3D Slicer, an open-source software for advanced 3D imaging, visualization, segmentation, and analysis [33]. This software also supports image-guided procedure planning and navigation. The analysis included thresholding, segmentation and volume measurement. The Threshold tool in 3D Slicer was used to define specific intensity ranges, isolating the lungs from surrounding structures. The Segment Editor module was employed to segment lung regions. The Segment Statistics provided volumetric measurements for each region.

The analysis workflow consisted of several steps. For each patient, the cranio-caudal range of analysis was defined by identifying the first (most apical) and last (most basal) axial slices on which the lungs were clearly visible. This range was determined using the RV dataset. This approach was chosen because the diaphragm is in its highest position at RV. This ensures that every selected slice for analysis contains lung parenchyma in both the RV and the corresponding TLC scans. This provides a consistent anatomical volume for comparison. Next, the lungs were divided into 20 slices from the apex to the base using equidistant planes, enabling detailed regional analysis. Segmentation was performed separately for lungs imaged at RV and TLC. For each slice, 3D Slicer automatically computed lung volumes at both RV and TLC. The air volume within each layer, or region’s total ventilation over a vital capacity maneuver, was determined as follows:

8 $${V}_{\mathrm{air,layer}}={V}_{\text{TLC, layer}}-{V}_{\text{RV, layer}}.$$

The method described was applied to six patients with varying degrees of disease progression. The air volumes obtained for each lung slice at RV and TLC were compared to identify regional variations and disease-specific patterns.

Segmentation results were reviewed by two radiologists to ensure accuracy and consistency across all datasets. Any discrepancies were resolved by consensus. Furthermore, the final datasets, including volumetric data, were exported in standardized formats (.csv) for statistical analysis.

### Computational implementation and uncertainty quantification

A computational model was developed in MATLAB [34] to integrate the alveolar and gravitational models described in the previous sections. The model is designed to simulate lung inflation under the influence of gravity and to assess the impact of physiological variability on its mechanical response.

The core of the model is a numerical script implemented in MATLAB that calculates the regional and total lung volumes for a given set of input parameters. As illustrated in the flowchart in Fig. 3, for each step in transpulmonary pressure $\Delta {P}_{tp},$ the script solves the equilibrium equation ${P}_{ta,i}=P(\lambda ({V}_{i}))$ for each of the 20 cranio-caudal layers to find the corresponding alveolar volume ${V}_{i}.$ The total lung volume is then calculated by summing the regional volumes, weighted by the alveolar number distribution (${N}_{i}$). All simulations were performed under the assumption of free breathing.

To account for the inherent variability in biological systems, we performed a UQ analysis. Key geometrical and material parameters were modeled as random variables rather than fixed constants. Table 1 details the probability distributions and moments for these uncertain parameters, which were derived from our previous work [29] and the literature [3, 35, 36]. Other parameters, such as the pleural pressure gradient, were treated as deterministic based on the model by Shi et al. [28]. An increment of $\Delta {P}_{tp}$ of $20$ ${cmH}_{2}O$ was made to correspond to inflation to TLC, while a decrement of $-7$ ${cmH}_{2}O$ to deflation to RV.

**Table 1:** Probability distributions for uncertain parameters used in the Monte Carlo analysis. All parameters were modeled using truncated normal distributions

Symbol	Description	Distribution	Moments (Mean, SD)	Units
*Healthy Parenchyma*				
${c}_{1}$	Elastin elasticity parameter	Truncated normal^a^	53.29, 6.93	cmH_2_O
${c}_{2}$	Collagen elasticity parameter	Truncated normal^a^	365.00, 60.70	cmH_2_O
${\chi }_{2}$	Collagen volume fraction	Truncated normal^b,c^	0.15, 0.07	[-]
*Fibrotic Parenchyma*				
${c}_{1}$	Elastin elasticity parameter	Truncated normal^a^	189.40, 44.50	cmH_2_O
${c}_{2}$	Collagen elasticity parameter	Truncated normal^a^	702.30, 170.20	cmH_2_O
${\chi }_{2}$	Collagen volume fraction	Truncated normal^b,c^	0.32, 0.16	[-]
*Shared Parameter*				
${V}_{0}$	Initial alveolar volume	Truncated normal^a,d^	420, 0.42	nL
$\epsilon$	Thickness-to-radius ratio	Deterministic	0.05^e^	[-]
${P}_{pl}$	Pleural pressure at FRC	Deterministic	−1 (base) to −10 (apex)^f^	cmH_2_O
$\Delta {P}_{tp}$	Transpulmonary pressure range	Deterministic	−7 (RV) to + 20 (TLC)^f^	cmH_2_O

^a^Distribution is truncated with lower and upper bounds of $[0,+\infty ]$. ^b^Distribution is truncated with lower and upper bounds of $[\mathrm{0.01,1}]$. ^c^Mean and standard deviations were calculated from the uniform range for consistency. ^d^Mean and standard deviation (10% coefficient of variation) from Ochs et al. [35]. ^e^Source [3]. ^f^Source [28]

Three simulation cases were introduced to compare the mechanics of healthy and fibrotic lungs. For healthy lungs, the material properties defined for healthy parenchyma in Table 1 were used. The corresponding collagen distribution, termed UH, is shown in the top-right panel of Fig. 2. For fibrotic lungs, two sub-cases were defined. The first sub-case simulates homogeneous fibrosis by applying the material properties of the fibrotic parenchyma uniformly across all 20 lung layers. The corresponding regionally uniform collagen volume fraction, termed UIPF, is represented in the middle-right panel of Fig. 2.

The second sub-case for fibrotic lungs models the distinct heterogeneity of IPF. It applies the material properties of the fibrotic parenchyma to the basal region (layers $1-14$) and the material properties of the healthy parenchyma to the apical region (layers $15-20$), creating a sharp mechanical gradient. The corresponding collagen distribution, termed SWIPF, is shown in the bottom-right panel of Fig. 2. The choice of layer 15 as the starting point for the apical layer is purely for illustrative purposes.

It is important to clarify that the constant and piecewise collagen fraction values shown in Fig. 2 for the UH, UIPF, and SWIPF scenarios represent the baseline or mean values used to define these cases. In the subsequent uncertainty quantification (UQ) analysis, however, these parameters were modeled as random variables to account for physiological variability, rather than being treated as fixed constants. As detailed in Table 1, the collagen fraction for healthy and fibrotic parenchyma, ${\chi }_{2}$, was modeled using a truncated normal distribution, sampling values around the established means. Thus, Fig. 2 illustrates the deterministic structure of the simulation scenarios, and Table 1 provides the full statistical description of the parameter uncertainty employed in the Monte Carlo analysis.

The introduction of a stepwise distribution of material properties is supported by three factors. First, the sharp transition mimics the spatial heterogeneity typical of pulmonary fibrosis, often characterized by patchy lesions rather than diffuse gradients. Next, the SWIPF case allows us to mechanically reproduce the specific peak in total regional ventilation observed in the clinical data of the patients, as illustrated in the forthcoming Sect. 3.3. Finally, the values of the material constants ${c}_{1}$ and ${c}_{2}$ were derived for healthy and fibrotic tissue from our previous work [29], but experimental data defining the continuous evolution of these parameters across a transition zone is unavailable. Using a discrete material assignment avoids introducing arbitrary, unvalidated interpolation functions for ${c}_{1}$ and ${c}_{2}$.

#### Uncertainty quantification methodology

To evaluate the impact of physiological variability on the model’s predictions, we conducted an UQ analysis using a Monte Carlo approach. The key uncertain parameters (${c}_{1},$ ${c}_{2},$ ${\chi }_{2}$ and ${V}_{0}$) were modeled as statistically independent random variables with specified probability distributions, as detailed in Table 1. We assumed statistically independent random variables for two reasons. First, the parameters were derived from different experimental modalities and literature sources, such as macroscopic pressure–volume curve fitting and stereological data. This makes it impossible to calculate their correlation directly. Second, the original data-fitting procedures did not provide sufficient information to estimate a full covariance matrix for the material parameters. While these properties may be physiologically correlated, assuming independence is reasonable in the absence of direct correlation data.

The elasticity parameters, ${c}_{1},$ ${c}_{2},$ the initial alveolar volume, ${V}_{0},$ and the collagen volume fractions for the healthy and fibrotic tissues were all modeled using truncated normal distributions. For the collagen volume fractions, the additional physical constraint ${\chi }_{2}\ge 0.01$ was imposed to avoid instability associated to the neo-Hookean strain energy model for elastin under imposed pressure. A total of $1000$ input parameter sets were generated by sampling from these distributions using the MATLAB *Statistics and Machine Learning Toolbox* [34]. For each of the three cases UH, UIPF, and SWIPF, the full lung model was then solved for each parameter set, and the resulting distributions of the outputs (TLC and RV) were analyzed to determine their mean, standard deviation, and 95% confidence intervals.

## Results

### Model validation against clinical data

To validate the model at a macroscopic level, we compared the outputs of the UQ analysis against the measured clinical data. Table 2 provides a comparison of the predicted and measured statistics for TLC and RV.

**Table 2:** Comparison of measured and predicted lung volumes at total lung capacity (TLC) and residual volume (RV) in healthy controls (H) and patients with idiopathic pulmonary fibrosis (IPF). The measured data are the mean $\pm$ standard deviation (SD). All values are in liters (L). %E is the percentage error in the predicted mean compared to the clinical cohort mean

Data Source	Description	TLC (L)	%E	RV (L)	%E
Measured (clinical data)	Healthy controls (H)	5.88 ± 0.66	−	2.06 ± 0.47	−
	IPF patients	3.95 ± 0.60	−	1.46 ± 0.38	−
Model predictions (UQ analysis)	Uniform healthy (UH)	5.40 ± 1.23	8.16	1.87 ± 0.19	9.22
	Uniform fibrotic (UIPF)	3.59 ± 0.49	9.11	1.96 ± 0.20	34.25
	Stepwise fibrotic (SWIPF)	3.89 ± 0.49	1.52	1.98 ± 0.20	35.62

The model predictions show strong agreement with clinically observed data, especially for TLC. As shown in Table 2, the predicted mean TLC for the SWIPF model (3.89 ± 0.49 L) differs from the mean observed in IPF patients (3.95 L) by only 1.52%. This result provides strong quantitative validation of the heterogeneous model. The model also successfully captures the restrictive characteristic of IPF pathology. The predicted mean TLC for the healthy (UH) model (5.40 ± 1.23 L) is significantly larger than for either fibrotic scenario: UIPF (3.59 L) and SWIPF (3.89 L). About the calculated variability, the predicted TLC for the UH model exhibits the largest standard deviation ($1.23$ L). This indicates a larger sensitivity to the full range of healthy anatomical and material properties. The standard deviations for the fibrotic models are smaller ($0.49$ L for both models), suggesting a less sensitive mechanical response in the fibrotic state to the variations of material and geometrical parameters.

While the model successfully captures the mechanics of lung inflation, it does not fully reproduce the trends observed at RV. The model predicts a slight increase in RV for the fibrotic cases compared to the healthy one, whereas the clinical data show a lower mean RV in IPF patients. This discrepancy is believed to be due to the closure of small airways that characterize fibrotic lungs, where the stiffening of the parenchyma can lead to premature collapse of small airways during exhalation, trapping less air and resulting in a lower RV. As our model focuses on alveolar mechanics and does not include an explicit model of airway dynamics, it cannot capture this phenomenon. This suggests that while alveolar properties are dominant factors in determining TLC, airway mechanics may play a much more significant role in setting the residual volume in IPF.

Finally, we observe that the simulated volumes at RV exhibit a nearly constant SD of approximately 0.2 L across all three scenarios. This is estimated to be a direct consequence of the modeling assumption, aligned with the approach of Shi et al. [28], where negative transalveolar pressures at RV are set to correspond to a unitary stretch to rule out non-physical alveolar collapse. This assumption makes the volume of most alveoli at RV independent of the tissue’s elastic properties. Consequently, the variability in the total predicted RV is mainly driven by the underlying anatomical uncertainty in the initial alveolar volume ${V}_{0}.$

### Pressure-volume responses under uncertainty

**Fig. 4:**
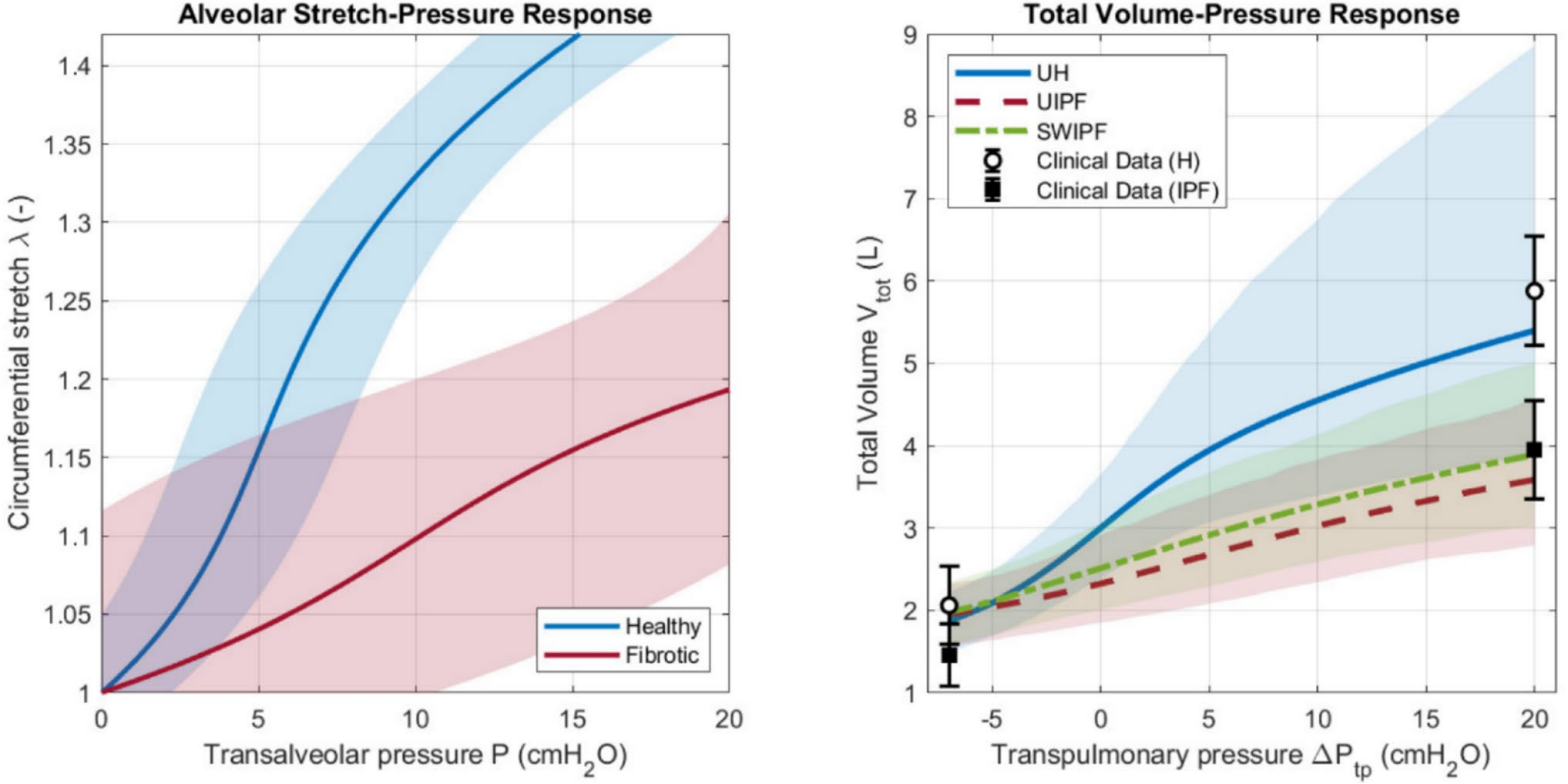
Predicted alveolar pressure-stretch and global pressure-volume responses with corresponding uncertainty intervals. Left panel: alveolar stretch-pressure response for healthy and fibrotic parenchyma. Solid lines represent the mean response from the parameter fit, and shaded regions represent the 95% prediction interval. Right panel: total volume-transpulmonary pressure responses from a 1000-sample Monte Carlo simulation. Lines represent the mean prediction for the UH, UIPF and SWIPF cases, while shaded regions represent the corresponding 95% confidence intervals. The error bars for the clinical data at total lung capacity (TLC) and residual volume (RV) are also shown

The left panel of Fig. 4 shows the alveolar stretch-pressure responses calculated using Eq. (1) with the parameters listed in Table 1 for healthy (H) and fibrotic (IPF) subjects along with their 95% prediction intervals, which represent uncertainty in the constitutive law derived from the original data fit [29]. As discussed in [29], elastin is expected to play a major role at low strain levels, while collagen becomes dominant at higher strain levels. For this reason, and due to the increased presence of collagen, which stiffens at large strains, IPF alveolar curves display altered compliance, with relative softness at low strain but increased stiffness at higher volumes. To investigate the effects of gravity and a non-uniform collagen distribution in the lungs, we plotted the global volume-pressure curves for healthy lungs with uniform (UH) collagen distribution (blue line), fibrotic lungs with uniform (UIPF) collagen distribution (red line) and fibrotic lungs with stepwise (SWIPF) collagen distribution (green line), represented in the right panel of Fig. 4. For visual comparison, the figure also shows the clinical data for RV and TLC (error bars). The healthy lung exhibits significantly higher compliance, achieving a total lung volume of around 5.40L at TLC (pressure value of 20 cm cmH_2_O), as indicated in Table 2. In contrast, both fibrotic models are stiffer: the UIPF model reaches 3.59 L and the SWIPF model reaches 3.89L at TLC, consistently with the reduced lung capacity characteristic of pulmonary fibrosis. All three curves converge to a similar residual volume (RV) of approximately 1.87L for UH and 1.96-1.98L for UIPF-SWIPF at the lowest transpulmonary pressure (−7 cmH_2_O). This indicates that, at low lung volumes, differences in tissue stiffness have a less significant impact on total lung volume, that is more influenced by the initial geometric and pressure conditions. A comparison of the curves for two fibrotic models shows that the SWIPF model (green curve) is slightly more compliant than the UIPF model (red curve). At any given positive pressure, the SWIPF lung achieves a slightly higher total volume. This is an expected result, as the SWIPF model is characterized by a more compliant apical region containing a 5% of collagen. This makes the lung less stiff than the uniformly dense fibrotic tissue containing 31% collagen that is assumed in the UIPF model.

### Regional volume distributions and variability

**Fig. 5:**
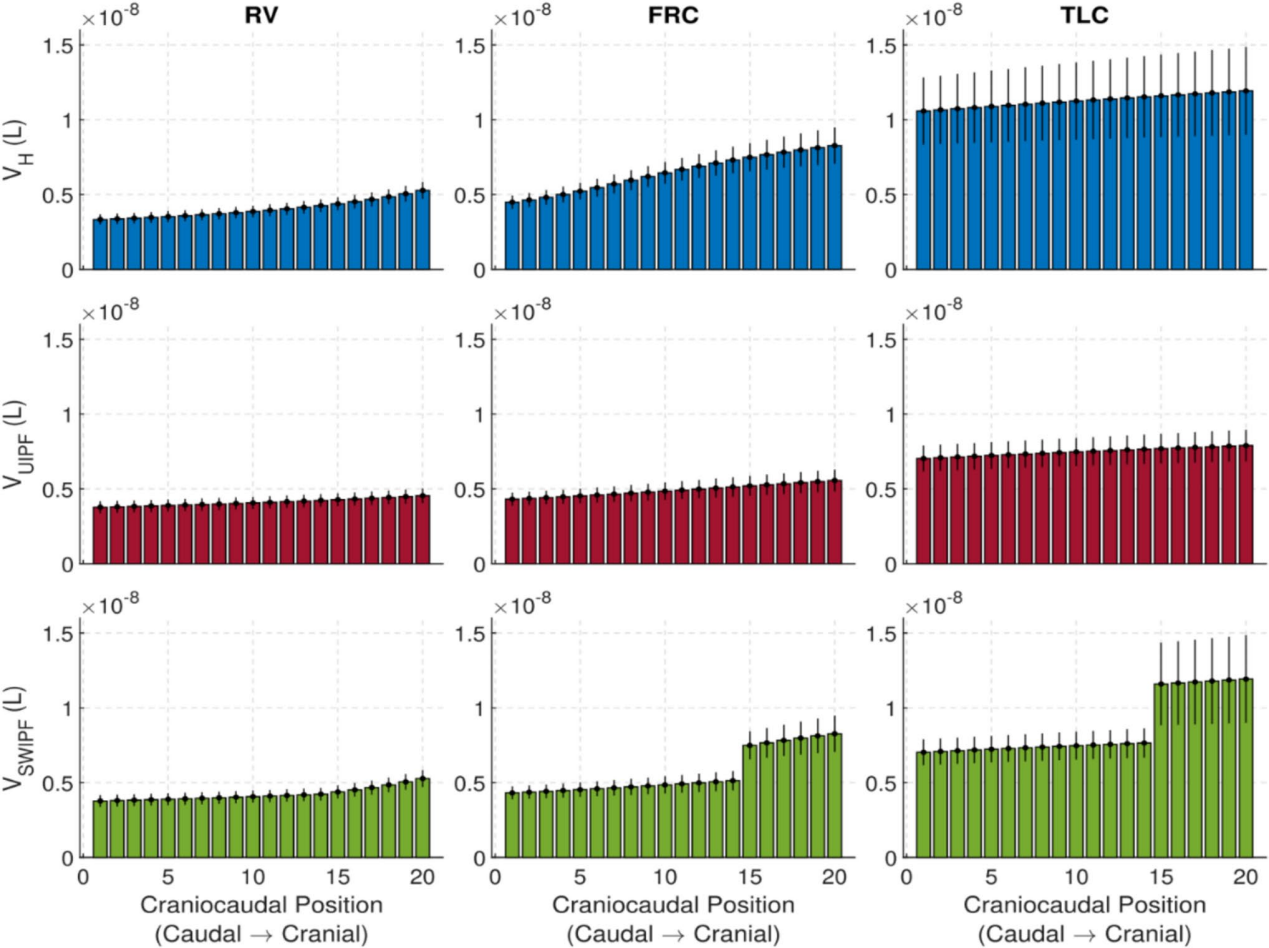
Comparison of the alveolar volume distributions in the 20 regions of the lung at RV, FRC and TLC for H (top panel), UIPF (middle panel) and SWIPF (bottom panel) models

In Fig. 5, we compare the mean predicted alveolar volume distributions at RV, FRC, and TLC in the presence of gravity for the three cases considered, UH, UIPF and SWIPF. The error bars represent the variability in the alveolar regional volume due to the combined uncertainty of the input parameters.

The regional alveolar volume distribution in the UH case is predicted to increase with the regional index. This is a gravity effect, due to the increasing of the applied pressure (and thus of the strain) from the base to the apex. As indicated by the error bars, the uncertainty is relatively uniform across the lungs and increases from RV to TLC. In fact, the absolute uncertainty in alveolar volume can be estimated as $\Delta V\approx 3{V}_{0}{\lambda }^{2}\Delta \lambda$ (cf. Equation (5)) and is thus amplified by the larger stretches occurring at TLC.

The rigidity of collagen, and thus of the fibrotic tissue, limits the expansion by reducing the alveolar volume in each lung region compared to a healthy lung. In addition, the non-uniform distribution of collagen affects the lung regions differently. By replacing the normal lung tissue with more rigid collagenous tissue, the expansion capacity at TLC is reduced, as observed in clinical practice. Lung regions comprising more collagen, and theoretically more impacted by fibrosis, maintain a limited volume, close to the resting volume. Conversely, regions with a smaller collagen content can achieve relatively higher volumes.

Accordingly, in the UIPF case, the mean alveolar volumes are reduced, with very little variation from base to apex. In the SWIPF case, the fibrotic basal region (layers $1-14$) exhibits low alveolar volumes with small variability. On the contrary, the compliant apical region (layers 15–20 ) undergoes compensatory hyperinflation. Notably, the variability in this apical region is also significantly larger, as shown by the larger error bars at TLC. This indicates that, in these areas, the mechanical response is more sensitive to the underlying material properties due to larger stretches and is also particularly vulnerable due to apical compensatory hyperinflation.

### Image analysis results

**Fig. 6:**
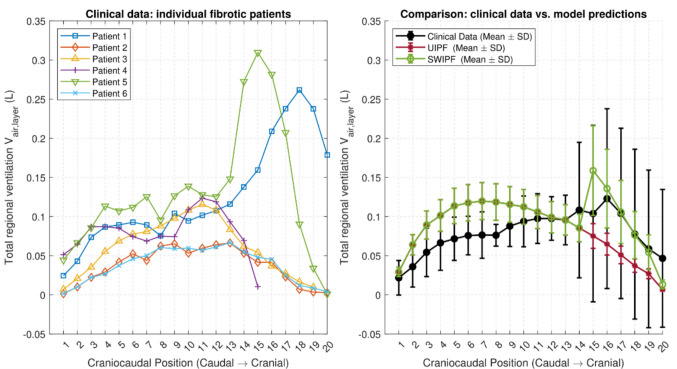
Distribution of total regional ventilation, ${V}_{air,layer}.$ Left panel: results obtained by analyzing CT scans of six fibrotic patients using Eq. (8). Right panel: comparison of the UQ analysis results and the statistical clinical results, averaged across six patients

Figure 6 illustrates the predicted total regional ventilation ${V}_{air,layer},$ as defined in Eq. (8), against corresponding data derived from the CT scans of six fibrotic patients. The left panel of the figure shows the total regional ventilation for each of the six fibrotic patients. While all patients exhibit a general pattern of reduced ventilation in the basal region, there is considerable variability in terms of the magnitude and location of the ventilation peak. Patients 2, 3 and 6 demonstrate increased ventilation in the central lung zones, with a decrease towards the apex and base. In contrast, patients 1 and 5 exhibit a broader ventilation peak shifted towards the apex (layers 17–19 and 14–16, respectively), likely due to the heterogeneous distribution of fibrotic tissue. These findings highlight the heterogeneity of the disease’s various manifestations. Patient 4 exhibits an anomaly in the apical region that merits specific mention. For this patient, the curve was only plotted up to layer 15. Beyond this point, the total regional ventilation computed using image analysis becomes negative. This implies that, at RV, there is more air in some alveolar regions than at TLC, which is physically impossible. This discrepancy is likely due to misalignment between the CT scans taken at RV and TLC, potentially caused by patient movement during scanning. Consequently, the slices being compared do not correspond to the same anatomical regions. To address this issue, an anatomical landmark should be used to ensure consistent comparisons of slices across the scans.

The right panel of Fig. 6 shows a comparison between the mean and standard deviation of the clinical data (black line and error bars) and the corresponding predictions for the UIPF (red line and error bars) and SWIPF (green line and error bars) modeling cases. In the basal region of the lung (layers 1–14), the UIPF and SWIPF models perfectly overlap, and are in good agreement with the mean of the clinical data. The UIPF models captures the increasing–decreasing trend exhibited by patients 2, 3 and 6. The same trend was observed by Petersson et al. [37] using quantitative Single Photon Emission Computed Tomography (SPECT) during continuous spontaneous breathing in upright healthy subjects and predicted for healthy lungs in normal and microgravity by Shi et al. [28] Fig. 6B.

The SWIPF model correctly predicts the ventilation peak occurring in patients 1 and 5 near the apical region (layers $15-20$), a probable direct result of the compensatory hyperinflation of the preserved parenchyma in that zone. To quantify the overall agreement between the predicted mean ventilation and the clinical mean across the entire cranio-caudal axis, we calculated the normalized root mean square error (NRMSE) with the error normalized by the range of the clinical data. The SWIPF model shows strong agreement with clinical data across the entire cranio-caudal axis, with an NRMSE of 19.75%. In contrast, while the UIPF model matches the data in the basal region, it deviates significantly in the apex. This results in a much larger overall error, with an NRMSE of 30.25%.

Furthermore, the SWIPF model also captures the magnitude of clinical variability in the region of hyperinflation. For each layer from 14 to 19, the model’s mean ± SD interval falls entirely within the corresponding clinical interval. For example, at layer 17, the model predicts a mean ± SD range of 0.106 ± 0.04 which is contained within the clinically observed range of 0.104 ± 0.109.

## Discussion

This study presents a computational model of lung mechanics that combines regional variations in material properties with gravitational effects. The model was tested through uncertainty quantification against clinical data on lung volumes (TLC and RV) and regional ventilation in both healthy and fibrotic cohorts. The overall agreement, particularly for the UIPF and SWIPF models, supports the importance of accounting for structural heterogeneity in IPF. The analysis also highlighted specific trends, such as the higher mechanical sensitivity of compliant regions and the distinct variability sources affecting TLC and RV. At the same time, the model relies on several assumptions and simplifications that delimit its scope and indicate directions for further improvement.

### Main findings

The main finding of our work is that accounting for cranio-caudal heterogeneity is essential to reproducing patient-specific lung mechanics. As shown in the right panel of Fig. 6, the SWIPF model’s piecewise collagen distribution correctly predicts the ventilation peak in patients 1 and 5 near the apical region, whereas the uniform UIPF model fails to do so. We link the peak to probable compensatory hyperinflation of the preserved parenchyma in that zone. The UQ analysis revealed that the apical region has the greatest mechanical variability, suggesting that it is particularly vulnerable due to a combination of high mean stretch and tissue sensitivity, which could drive IPF progression via local mechanical triggers, such as the “squishy effect” proposed in [38] and theoretically studied in [20].

The UQ analysis reveals the primary drivers of variability in the model. The predicted RV exhibits a nearly constant standard deviation of approximately 0.19 L across the three proposed mechanical scenarios, H, UIPF, and SWIPF. This minor sensitivity to different tissue elastic properties (${c}_{1}$, ${c}_{2}$, and ${\chi }_{2}$) suggests that RV variability could be dominated by uncertainty in the single shared parameter: the initial alveolar volume ${V}_{0}$. This is believed to be a direct consequence of the modeling assumption at low lung volumes, when most alveoli experience unitary stretch, rendering their volume independent of tissue stiffness. In contrast, the predicted TLC shows variable standard deviations across the three simulations, e.g., 1.69 L for H versus 0.56 L for SWIPF. This demonstrates that, as expected, the model’s output is more sensitive to the complex, nonlinear interplay of all the material properties that govern the tissue’s stiffening response at high lung volumes.

### Mechanical insights and clinical implications

Our results provide strong qualitative support for the “mechanical vicious cycle” [4, 6, 8, 39]. The ventilation peak and hyperinflation predicted at the boundary between healthy and fibrotic tissue are thought to act as mechanical stimuli that can initiate and perpetuate pro-fibrotic cellular responses like TGF-β1 activation [7, 10]. This drives disease progression from the base to the apex. Furthermore, the predicted ventilation patterns are in good qualitative agreement with experimental observations from functional lung imaging studies [28, 37], an occurrence that provides strong external validation for the present model. Future studies could couple our model with ABMs to mechanically explain the spatial patterns of IPF progression [16–18]. This validated framework could be the first step in developing patient-specific computational models based on individual CT data and enable personalized predictions of disease progression or the mechanical effects of ventilation strategies.

### Limitations of the model

Several assumptions underline the proposed framework. First, the mechanical model inherits many simplifications from earlier approaches and introduces new ones. The lung is modeled as a collection of independent alveoli represented as thin spherical membranes, which neglects structural interdependence and load transfer between adjacent alveoli. This simplification may limit accuracy in predicting local stress distributions and alveolar stability. The pressure–stretch relationship was also simplified. Material parameters were estimated from macroscopic pressure–volume curves, which implicitly capture both elastic tissue response and surface tension, but do not allow a separate analysis of these effects, as done for example in [40]. As a result, surfactant dynamics and hysteresis could not be investigated explicitly. Finally, the model considers purely elastic behavior, neglecting viscoelasticity, which is relevant for dynamic breathing and stress relaxation.

Second, pleural pressure was assumed to vary linearly from base to apex, while it is influenced by additional anatomical and physiological factors, such as chest wall geometry and mediastinal weight. Horizontal heterogeneity of pleural pressure was also neglected. For both healthy and fibrotic lungs, identical distributions of alveolar number, pleural pressure, and transpulmonary pressure were assumed. In lung fibrosis, however, alveolar loss, increased stiffness, and altered surfactant function modify these distributions [4, 41]. Since detailed patient-specific data is not currently available, these aspects remain a limitation.

Another limitation of this study is the use of an upright pleural pressure gradient when comparing model predictions with CT scans acquired in the supine position. We adopted the canonical upright gradient because a validated posture-specific pleural pressure distribution for fibrotic lungs is not available, and because our aim was to investigate fundamental mechanical trends rather than reproduce posture-dependent absolute pressure fields.

Validation against imaging data presented additional challenges. Lung segmentation was performed manually, which introduces the possibility of operator-related errors, for instance by misclassifying air or fluid regions. Segmentation accuracy depends strongly on the operator’s expertise in identifying anatomical boundaries. Patient positioning during TLC or RV scans also affects the reliability of volumetric measurements. Future studies could reduce these sources of error by using anatomical markers or automated three-dimensional segmentation methods. Patient-specific differences remain another source of variability, as the current UIPF and SWIPF parameterizations may not capture the full heterogeneity of IPF lungs. Finally, horizontal gradients in material properties were not considered in the present framework.

### Future directions

The framework provides a foundation for developing more advanced, multi-scale, and patient-specific models. Extending the current approach to a three-dimensional alveolar network would enable the simulation of load transfer between alveoli and improve strain localization prediction near fibrotic lesions. Personalized, CT-based maps of fibrosis distribution could be incorporated to assign local material properties and predict ventilation in individual patients. This would enable direct validation against their functional imaging data.

Another major area of development could be integrating more sophisticated constitutive behaviors to capture the multiphysics nature of the lung parenchyma and move beyond the current nonlinear elastic model [3, 19, 42, 43]. Incorporating biological growth and remodeling is essential to simulating fibrosis progression over time [16–18]. Similarly, accounting for fluid-solid interactions through a poroelastic formulation would be crucial for investigating transient phenomena [25–27, 44]. To generalize the current model’s symmetry assumptions, these advanced physics could be coupled with rigorous computational upscaling techniques, such as asymptotic homogenization, to derive effective behaviors from microstructurally detailed representative volume elements [23, 24]. Furthermore, a formal global sensitivity analysis, using efficient methods like polynomial chaos expansions, could be performed to rigorously quantify the influence of each input parameter on the model’s predictions [45, 46].

Finally, the predictive power of these advanced models must be grounded in experimental data. Personalized CT-based maps of fibrosis distribution could be used to assign local material properties, enabling direct validation of predicted ventilation patterns against functional imaging data from individual patients. On a smaller scale, micromechanical measurements from techniques such as atomic force microscopy on lung ECM components are essential for calibrating the parameters of these more sophisticated constitutive models. Taken together, these parallel developments in geometric complexity, physical realism, and experimental validation will advance this framework.

## Conclusion

In this study, we developed and validated a biomechanical model that combines the effects of gravity and a non-uniform cranio-caudal distribution of tissue elasticity and collagen content for the first time. This model simulates the mechanics of the fibrotic lung. Our results demonstrate that structural heterogeneity, in conjunction with gravity, may lead to regional ventilation peaks, a phenomenon supported by patient-specific data.

This work is relevant because it provides a validated framework that could serve as a foundation for future studies investigating disease progression or the impact of therapeutic interventions, such as mechanical ventilation. Ultimately, our study underscores the importance of accounting for structural heterogeneity in biomechanical models to improve our understanding of complex lung diseases.
